# Consistency and Stability of Motor Subtype Classifications in Patients With *de novo* Parkinson’s Disease

**DOI:** 10.3389/fnins.2021.637896

**Published:** 2021-03-01

**Authors:** Jingru Ren, Chenxi Pan, Yuqian Li, Lanting Li, Ping Hua, Ligang Xu, Li Zhang, Wenbin Zhang, Pingyi Xu, Weiguo Liu

**Affiliations:** ^1^Department of Neurology, The Affiliated Brain Hospital of Nanjing Medical University, Nanjing, China; ^2^Department of Geriatrics, The Affiliated Brain Hospital of Nanjing Medical University, Nanjing, China; ^3^Department of Neurosurgery, The Affiliated Brain Hospital of Nanjing Medical University, Nanjing, China; ^4^Department of Neurology, First Affiliated Hospital of Guangzhou Medical University, Guangzhou, China

**Keywords:** *de novo* Parkinson’s disease, motor subtypes, non-motor symptoms, tremor-dominant, postural instability and gait difficulty, akinetic-rigid

## Abstract

**Objective:**

Patients with Parkinson’s disease (PD) are commonly classified into subtypes based on motor symptoms. The aims of the present study were to determine the consistency between PD motor subtypes, to assess the stability of PD motor subtypes over time, and to explore the variables influencing PD motor subtype stability.

**Methods:**

This study was part of a longitudinal study of *de novo* PD patients at a single center. Based on three different motor subtype classification systems proposed by Jankovic, Schiess, and Kang, patients were respectively categorized as tremor-dominant/indeterminate/postural instability and gait difficulty (TD/indeterminate/PIGD), TD_*S*_/mixed_*S*_/akinetic-rigid_*S*_ (ARS), or TD_*K*_/mixed_*K*_/AR_*K*_ at baseline evaluation and then re-assessed 1 month later. Demographic and clinical characteristics were recorded at each evaluation. The consistency between subtypes at baseline evaluation was assessed using Cohen’s kappa coefficient (κ). Additional variables were compared between PD subtype groups using the two-sample *t*-test, Mann–Whitney *U*-test or Chi-squared test.

**Results:**

Of 283 newly diagnosed, untreated PD patients, 79 were followed up at 1 month. There was fair agreement between the Jankovic, Schiess, and Kang classification systems (κ_*S*_ = 0.383 ± 0.044, κ_*K*_ = 0.360 ± 0.042, κ_*SK*_ = 0.368 ± 0.038). Among the three classification systems, the Schiess classification was the most stable and the Jankovic classification was the most unstable. The non-motor symptoms questionnaire (NMSQuest) scores differed significantly between PD patients with stable and unstable subtypes based on the Jankovic classification (*p* = 0.008), and patients with a consistent subtype had more severe NMSQuest scores than patients with an inconsistent subtype.

**Conclusion:**

Fair consistency was observed between the Jankovic, Schiess, and Kang classification systems. For the first time, non-motor symptoms (NMSs) scores were found to influence the stability of the TD/indeterminate/PIGD classification. Our findings support combining NMSs with motor symptoms to increase the effectiveness of PD subtypes.

## Introduction

The clinical presentations of Parkinson’s disease (PD) are considerably heterogeneous (Armstrong and Okun, 2020). It is difficult to delineate if such variability between PD patients reflects the various manifestations of a unitary disease or represents different disease subtypes driven by underlying pathological and pathophysiological distinctions (Calne, 1989). The solution to this problem is generally thought to be through the use of PD subtypes.

Classification of PD subtypes is based on empirical clinical observations of prominent motor symptoms. At present, the recognized PD motor subtype classifications, including tremor-dominant/indeterminate/postural instability and gait difficulty (TD/indeterminate/PIGD) put forward by Jankovic and two classifications of the TD/mixed/akinetic-rigid (AR) classification system proposed by Schiess and Kang (Jankovic et al., 1990; Schiess et al., 2000; Kang et al., 2005), are widely used (Guan et al., 2017; Choi et al., 2018; Erro et al., 2019; Lian et al., 2019; Polychronis et al., 2019; Ren et al.,2020a,b). Although numerous additional subtypes based on data-driven approaches have been subsequently proposed (Graham and Sagar, 1999; van Rooden et al., 2010; Fereshtehnejad et al., 2015), the above three classifications occupy a dominant position in clinical practice and scientific research and have entered the conventional lexicon of clinicians.

Accurately subtyping patients is an indispensable step for research on disease mechanisms, for prodromal and clinical trial design and, in particular, for the development of tailored treatments. However, the terminology used to describe PD motor subtypes has overlapping boundaries, which can result in confusion and lead to an extensively inaccurate literature (Kotagal, 2016). A recent study found that the consistency between the Jankovic classification and Schiess classification is poor (Erro et al., 2019). In addition, temporal instability of these two classifications in *de novo* PD patients has been reported (Simuni et al., 2016; Erro et al., 2019).

To our knowledge, studies of the consistency of the Kang, Jankovic, and Schiess classifications, or of the stability of the Kang classification alone, have not been performed. It is also unknown whether factors besides the disease course influence the stability of motor subtypes (Simuni et al., 2016; Eisinger et al., 2017, 2020). To address these gaps, this study aimed to examine the consistency between the TD/indeterminate/PIGD (Jankovic classification) and two TD/mixed/AR classification systems (Schiess and Kang classification) in 283 *de novo* PD patients at baseline and then determine the stability of the three classification systems over 1-month follow-up in a subgroup of 79 patients. In addition, we aimed to identify variables that affected the stability of the motor subtypes.

## Methods

### Participants

This study was part of a longitudinal study of *de novo* PD patients conducted at the Department of Neurology, Affiliated Brain Hospital of Nanjing Medical University from January 2012 to October 2020. All patients who came to the clinic were examined by a movement disorder specialist. A total of 283 *de novo* PD patients fulfilled all the study inclusion and exclusion criteria, of which 27.9% had evaluation data for the first month of follow-up. The inclusion criteria were: (1) the presence of bradykinesia plus an additional clinical motor sign, namely resting tremor, rigidity, or postural instability; (2) be newly diagnosed with PD based on the United Kingdom Parkinson’s Disease Society Brain Bank clinical diagnostic criteria (Gibb and Lees, 1988); (3) be untreated; (4) have early- or middle-stage PD (modified H-Y stage ≤3); (5) have more than a 30% improvement rate in the Unified Parkinson’s Disease Rating Scale (UPDRS) part III scores by the standard acute levodopa challenge test; (6) receive follow-up through hospital visits for at least 1 year, especially for motor symptoms assessment; and (7) have detailed clinical evaluation information available. The exclusion criteria were: (1) the presence of atypical or secondary Parkinsonism disorders; (2) previous brain magnetic resonance imaging (MRI) scans with obvious clinically significant lesions; and (3) severe chronic diseases.

This study was approved by the Medical Ethics Committee of the Affiliated Brain Hospital of Nanjing Medical University (2011-KY003, 2015-KY030, and 2019-KY019-01) and conducted in accordance with the ethical standards outlined in the Helsinki Declaration of 1964. All subjects provided written informed consent before participating in this study.

### Clinical Evaluation

All baseline clinical evaluations were performed before initiation of dopaminergic replacement therapy (DRT), which includes levodopa (L-DOPA) and dopamine receptor agonists (DAs). After starting DRT, 1-month follow-up evaluations were performed in the “practically defined OFF state” (Langston et al., 1992) and the levodopa equivalent daily dose (LEDD) was calculated (Tomlinson et al., 2010). Patients’ demographic and clinical characteristics were recorded at the baseline and 1-month follow-up visits. Demographic data included age, gender, formal education in years, age at onset, and years of PD symptom onset duration. Motor dysfunction and disease severity were assessed by the UPDRS part II, III and the modified Hoehn and Yahr (H-Y) stages, respectively. General cognition, mood and sleep were measured using the Mini-Mental State Examination (MMSE), Montreal Cognitive Assessment (MoCA), Hamilton Depression Scale (HAMD), Hamilton Anxiety Scale (HAMA), and the Parkinson Disease Sleep Scale (PDSS), respectively. Non-motor symptoms (NMSs) were assessed using the non-motor symptoms questionnaire (NMSQuest) (Chaudhuri et al., 2006).

### Subtype Classification

UPDRS items selected according to a published formula were used to classify patients as TD/indeterminate/PIGD or TD/mixed/AR motor subtypes (Table 1; Jankovic et al., 1990; Schiess et al., 2000; Kang et al., 2005). For clarity, TD_*S*_/mixed_*S*_/AR_*S*_ refers to the classification proposed by Schiess whereas TD_*K*_/mixed_*K*_/AR_*K*_ refers to the classification proposed by Kang.

**TABLE 1:** Items used for the tremor-dominant/indeterminate/postural instability and gait difficulty (TD/indeterminate/PIGD) and TD/mixed/akinetic-rigid (AR) classifications.

TD/indeterminate/PIGD (Jankovic classification)	TD_*S*_/mixed_*S*_/AR_*S*_ (Schiess classification)	TD_*K*_/mixed_*K*_/AR_*K*_ (Kang classification)
*Tremor score*	*Tremor score*	*Tremor score*
UPDRS-II	UPDRS-II	UPDRS-II
2.16 Tremor	2.16 Tremor RUE	
	2.16 Tremor LUE	
UPDRS-III	UPDRS-III	UPDRS-III
3.20 Rest tremor face	3.20 Rest tremor face	3.20 Rest tremor face
3.20 Rest tremor RUE	3.20 Rest tremor RUE	3.20 Rest tremor RUE
3.20 Rest tremor LUE	3.20 Rest tremor LUE	3.20 Rest tremor LUE
3.20 Rest tremor RLE	3.20 Rest tremor RLE	3.20 Rest tremor RLE
3.20 Rest tremor LLE	3.20 Rest tremor LLE	3.20 Rest tremor LLE
3.21 Action tremor RUE	3.21 Action tremor RUE	3.21 Action tremor RUE
3.21 Action tremor LUE	3.21 Action tremor LUE	3.21 Action tremor LUE
*PIGD score*	*AR score*	*AR score*
UPDRS-II	UPDRS-II	UPDRS-II
2.13 Falling		
2.14 Freezing		
2.15 Walking		
UPDRS-III	UPDRS-III	UPDRS-III
3.29 Gait	3.22 Rigidity neck	3.22 Rigidity neck
3.30 Postural stability	3.22 Rigidity RUE	3.22 Rigidity RUE
	3.22 Rigidity LUE	3.22 Rigidity LUE
	3.22 Rigidity RLE	3.22 Rigidity RLE
	3.22 Rigidity LLE	3.22 Rigidity LLE
	3.23 Finger taps	3.23 Finger taps
	3.24 Hand movements	3.24 Hand movements
	3.27 Arising from chair	3.25 Rapid alternating movements of hands
	3.28 Posture	3.26 Leg Agility
	3.29 Gait	3.27 Arising from chair
	3.30 Postural stability	3.31 Body bradykinesia
	3.31 Body bradykinesia	

*The table lists the UPDRS items used to calculate each patient’s tremor score, PIGD score, and AR score in the different subtype classification systems. Specifically, the ratio of mean tremor score to mean PIGD score is used to divide motor subtypes into TD (ratio ≥ 1.5), indeterminate (1.0 < ratios < 1.5), and PIGD (ratio ≤ 1). Similarly, the ratio of mean tremor score to mean AR score divides motor subtypes into TD_*S*_ (ratio > 1), mixed_*S*_ (0.8 < ratios ≤ 1.0), and AR_*S*_ (ratio ≤ 0.8). In addition, the ratio of tremor score (sum of items 20–21 divided by 4) to AR score (sum of items 22–27 and 31 divided by 15) divides motor subtypes into TD_*K*_ (ratio > 1), mixed_*K*_ (0.8 ≤ ratios ≤ 1.0), and AR_*K*_ (ratio < 0.8). UPDRS, unified Parkinson’s disease rating scale; RUE, right upper extremity; LUE, left upper extremity; RLE, right lower extremity; LLE, left lower extremity.*

### Statistical Analysis

Statistical analysis was conducted using IBM SPSS software version 25.0. The level of statistical significance was *P* < 0.05. The Kolmogorov-Smirnov test was used to assess the normality of the data. Differences in gender between groups were assessed using the chi-square test. Differences in baseline demographic and clinical variables other than gender between patients with and without 1-month follow-up assessment and between patients with stable and unstable subtypes in the TD/indeterminate/PIGD classification system were assessed using the two-sample *t*-test when the data were normally distributed; otherwise, the Mann–Whitney *U*-test was used. Cohen’s kappa coefficient (κ) was used to analyze agreement between the Jankovic, Schiess, and Kang classification systems at baseline. A κ value < 0.00 represents poor agreement, 0.00–0.20 represents slight agreement, 0.21–0.40 represents fair agreement, 0.41–0.60 represents moderate agreement, 0.61–0.80 represents substantial agreement and 0.81–1.00 represents almost perfect agreement (Landis and Koch, 1977).

## Results

### Demographic and Clinical Characteristics

Table 2 presents patients’ baseline demographic and clinical characteristics. Of the 283 PD patients enrolled in this study, clinical evaluation data at 1-month follow-up were available for 79 (27.9%). There were no significant differences in terms of demographic characteristics (age, gender, education, age at PD onset, and duration of symptom onset), motor symptoms (UPDRS part II, UPDRS part III, and modified H-Y stage), or NMSs (MMSE, MoCA, HAMD, HAMA, PDSS, and NMSQuest) between patients with and without 1-month follow-up data, indicating that the high drop-out rate did not affect the results. In addition, the demographic and clinical characteristics of the 79 PD patients at 1-month follow-up are provided in Supplementary Table 1. At 1-month follow-up, LEDD for 79 patients was 74.5 ± 4.2 mg.

**TABLE 2:** Baseline demographic and clinical characteristics.

Variables	Baseline (*n* = 283) (100.0%)	With 1-month (*n* = 79) (27.9%)	Without 1-month (*n* = 204) (72.1%)	*p*-value
Age (years)	61.5 ± 8.9	60.5 ± 7.3	61.9 ± 9.5	0.186
Gender (male)	141 (49.8%)	39 (49.4%)	102 (50.0%)	0.924
Formal education (years)	8.9 ± 4.7	9.6 ± 3.7	8.6 ± 5.0	0.259
Age at onset (years)	59.2 ± 8.9	58.5 ± 7.4	59.5 ± 9.4	0.319
Duration of symptom onset (years)	2.3 ± 2.3	2.1 ± 1.5	2.4 ± 2.5	0.859
UPDRS part II	9.3 ± 5.5	8.3 ± 4.5	9.7 ± 5.8	0.062
UPDRS part III	22.5 ± 11.6	22.6 ± 11.7	22.4 ± 11.7	0.890
Modified H-Y stage	1.6 ± 0.6	1.5 ± 0.5	1.6 ± 0.6	0.764
MMSE	26.7 ± 3.9	27.0 ± 3.1	26.6 ± 4.1	0.758
MoCA	22.5 ± 5.2	22.5 ± 4.0	22.5 ± 5.6	0.283
HAMD	10.1 ± 8.3	9.0 ± 5.2	10.5 ± 9.2	0.948
HAMA	7.8 ± 6.5	6.4 ± 4.1	8.3 ± 7.2	0.171
PDSS	121.7 ± 24.2	123.7 ± 26.4	120.9 ± 23.3	0.154
NMSQuest	8.6 ± 4.6	8.4 ± 3.6	8.7 ± 5.0	0.973

*Data are presented as the mean ± SD and *n* (%). Comparisons of demographic and clinical features at baseline between patients with and without 1-month follow-up assessments. Univariate *p*-values were calculated using two-sample *t*-test, Mann–Whitney *U*-test, or Chi-squared test. UPDRS, unified Parkinson’s disease rating scale; H-Y, Hoehn and Yahr; MMSE, Mini-mental state examination; MoCA, Montreal Cognitive Assessment; HAMD, Hamilton Depression Scale; HAMA, Hamilton Anxiety Scale; PDSS, Parkinson disease sleep scale; NMSQuest, non-motor symptoms questionnaire.*

### Baseline Consistency Between the Jankovic, Schiess, and Kang Classification Systems

At baseline, 83 (29.3%) patients were classified as TD, 159 (56.2%) as PIGD and 41 (14.5%) as indeterminate in TD/indeterminate/PIGD subtypes of the Jankovic classification. Schiess and Kang have each proposed method for categorizing patients into TD/mixed/AR subtypes. Using the Schiess classification, the majority subtype was AR_*S*_ with 213 cases (75.3%), followed by 49 (17.3%) TD_*S*_ cases and 21 (7.4%) mixed_*S*_ cases. Using the classification proposed by Kang, there were 124 (43.8%) TD_*K*_ cases, 135 (47.7%) AR_*K*_ cases, and 24 (8.5%) mixed_*K*_ cases. There was fair agreement between the Jankovic, Schiess, and Kang classification systems (κ_*S*_ = 0.383 ± 0.044, κ_*K*_ = 0.360 ± 0.042, κ_*SK*_ = 0.368 ± 0.038) (Table 3).

**TABLE 3:** Baseline consistency between the Jankovic, Schiess, and Kang classification systems.

		Schiess classification			Kang classification		
Baseline *N* = 283 (100%)		TD_*S*_ 49 (17.3%)	AR_*S*_ 213 (75.3%)	Mixed_*S*_ 21 (7.4%)	TD_*K*_ 124 (43.8%)	AR_*K*_ 135 (47.7%)	Mixed_*K*_ 24 (8.5%)
**Jankovic classification**	**TD 83** **(29.3%)**	37 44.6%/75.5%	33 39.8%/15.5%	13 15.7%/61.9%	64 77.1%/51.6%	14 16.9%/10.4%	5 6.0%/20.8%
	**PIGD 159** **(56.2%)**	5 3.1%/10.2%	151 95.0%/70.9%	3 1.9%/14.3%	32 20.1%/25.8%	110 69.2%/81.5%	17 10.7%/70.8%
	**Indeterminate 41** **(14.5%)**	7 17.1%/14.3%	29 70.7%/13.6%	5 12.2%/23.8%	28 68.3%/22.6%	11 26.8%/8.1%	2 4.9%/8.3%

*In each cell, the number of patients observed and their proportions in rows and columns are shown. For example, in the TD/indeterminate/PIGD and TD_*S*_/mixed_*S*_/AR_*S*_ classification system, 37 patients were classified as TD subtype, which accounted for 44.6% of the 83 TD patients and 75.5% of the 49 TD_*S*_ patients. There was fair agreement between the TD/indeterminate/PIGD and TD_*S*_/mixed_*S*_/AR_*S*_ classification systems (κ_*S*_ = 0.383 ± 0.044). There was fair agreement between the TD/indeterminate/PIGD and TD_*K*_/mixed_*K*_/AR_*K*_ classification systems (κ_*K*_ = 0.360 ± 0.042). There was fair agreement between the TD_*S*_/mixed_*S*_/AR_*S*_ and TD_*K*_/mixed_*K*_/AR_*K*_ classification systems (κ_*SK*_ = 0.368 ± 0.038). TD, tremor-dominant; PIGD, postural instability and gait difficulty; AR, akinetic-rigid.*

### Stability of the Jankovic, Schiess, and Kang Classification Systems

Changes in the Jankovic, Schiess, and Kang classification systems from baseline to 1-month follow-up among the 79 patients with longitudinal data are presented in Table 4. Using the Jankovic classification, 12 (54.5%) of the initially classified TD subtype cases and 37 (72.5%) of the initially classified PIGD subtype cases remained stable at 1-month follow-up. Using the Schiess classification, all of the initially classified TD_*S*_ subtype cases and 54 (83.1%) of the AR_*S*_ subtype cases were consistent at 1-month follow-up. Using the Kang classification, 15 (75.0%) of the initially classified TD_*K*_ cases and 35 (68.6%) of the initially classified AR_*K*_ cases were stable at 1-month follow-up. Among the three motor subtype classification systems, in the Schiess classification, the number of PD patients with stable motor subtypes from baseline to 1-month follow-up (65 cases in total, including 11 cases of TD_*S*_ subtype and 54 cases of AR_*S*_ subtype) was the largest, while in the Jankovic classification, the number of PD patients with stable motor subtypes (49 cases in total, including 12 cases of TD subtype and 37 cases of PIGD subtype) was the smallest. Therefore, the Schiess classification was the most stable and thus might be the most valid motor classification system, whereas the Jankovic classification was the most unstable.

**TABLE 4:** Stability of the Jankovic, Schiess, and Kang classification systems from baseline to 1-month follow-up.

		1-Month follow up				
Jankovic classification	Baseline	TD	PIGD	Indeterminate	Stable	Unstable
TD, *N* (Row%)	22(27.8%)	12(54.5%)	6(27.3%)	4(18.2%)	12(54.5%)	10(45.5%)
PIGD, *N* (Row%)	51(64.6%)	7(13.7%)	37(72.5%)	7(13.7%)	37(72.5%)	14(27.5%)
Indeterminate, *N* (Row%)	6(7.6%)	6(100.0%)	0(0.0%)	0(0.0%)	0(0.0%)	6(100.0%)
Total, *N* (Row%)	79(100.0%)	25(31.6%)	43(54.4%)	11(13.9%)	49(62.0%)	30(38.0%)

**Schiess classification**		**TD_*S*_**	**AR_*S*_**	**Mixed_*S*_**		

TD_*S*_, *N* (Row%)	11(13.9%)	11(100.0%)	0(0.0%)	0(0.0%)	11(100.0%)	0(0.0%)
AR_*S*_, *N* (Row%)	65(82.3%)	6(9.2%)	54(83.1%)	5(7.7%)	54(83.1%)	11(16.9%)
Mixed_*S*_, *N* (Row%)	3(3.8%)	2(66.7%)	1(33.3%)	0(0.0%)	0(0.0%)	3(100.0%)
Total, *N* (Row%)	79(100.0%)	19(24.1%)	55(69.6%)	5(6.3%)	65(82.3%)	14(17.7%)

**Kang classification**		**TD_*K*_**	**AR_*K*_**	**Mixed_*K*_**		

TD_*K*_, *N* (Row%)	20(25.3%)	15(75.0%)	4(20.0%)	1(5.0%)	15(75.0%)	5(25.0%)
AR_*K*_, *N* (Row%)	51(64.6%)	9(17.6%)	35(68.6%)	7(13.7%)	35(68.6%)	16(31.4%)
Mixed_*K*_, *N* (Row%)	8(10.1%)	4(50.0%)	4(50.0%)	0(0.0%)	0(0.0%)	8(100.0%)
Total, *N* (Row%)	79(100.0%)	28(35.4%)	43(54.4%)	0(0.0%)	50(63.3%)	29(36.7%)

*In each cell, the number of patients observed and their proportions in rows are shown. For example, for the Jankovic classification, at baseline 22 (27.8%) patients were classified as TD, 51 (64.6%) patients were classified as PIGD, and 6 (7.6%) patients were classified as indeterminate. At 1-month follow up, 12 (54.5%) of the original TD subtype remained TD and 37 (72.5%) of the original PIGD subtype remained PIGD. Therefore, the motor subtypes of 49 (62.0%) patients with PD remained stable in the Jankovic classification from baseline to 1-month follow-up. TD, tremor-dominant; PIGD, postural instability and gait difficulty; AR: akinetic-rigid.*

As the TD/indeterminate/PIGD classification system proposed by Jankovic was the most unstable, the baseline demographics and clinical characteristics of patients with stable and unstable subtypes were compared in this classification system to further analyze potential factors affecting the stability of PD motor subtypes. No significant differences in demographic or clinical characteristics between patients with stable and unstable subtypes, other than NMSQuest scores (*p* = 0.008), were observed. Patients with consistent subtypes had more severe NMSQuest scores than patients with inconsistent subtypes (Table 5).

**TABLE 5:** Comparisons of baseline demographic and clinical characteristics between patients with stable and unstable subtypes in the Jankovic classification.

Variables	Stable (*n* = 49) (62.0%)	Unstable (*n* = 30) (38.0%)	*p*-value
Age (years)	61.3 ± 7.3	59.3 ± 7.2	0.230
Gender (male)	22 (44.9%)	17 (56.7%)	0.310
Formal education (years)	9.9 ± 3.4	9.1 ± 4.2	0.446
Age at onset (years)	59.2 ± 7.4	57.2 ± 7.3	0.248
Duration of symptom onset (years)	2.1 ± 1.5	2.0 ± 1.5	0.678
UPDRS part II	8.8 ± 4.6	7.5 ± 4.2	0.169
UPDRS part III	23.6 ± 11.5	21.1 ± 12.0	0.223
Modified H-Y stage	1.6 ± 0.5	1.5 ± 0.5	0.321
MMSE	27.0 ± 3.2	27.1 ± 2.8	0.627
MoCA	22.6 ± 4.1	22.3 ± 3.9	0.715
HAMD	9.7 ± 5.1	7.9 ± 5.2	0.137
HAMA	7.0 ± 4.3	5.5 ± 3.8	0.154
PDSS	119.8 ± 30.0	130.2 ± 17.6	0.108
NMSQuest	9.2 ± 3.4	7.1 ± 3.5	**0.008**

*Data are presented as the mean ± SD and *n* (%). Statistically significant (*p* < 0.05) values are indicated in bold. Univariate *p*-values were calculated using two-sample *t*-test, Mann–Whitney *U*-test, or Chi-squared test. UPDRS, unified Parkinson’s disease rating scale; H-Y, Hoehn and Yahr; MMSE, Mini-mental state examination; MoCA, Montreal Cognitive Assessment; HAMD, Hamilton Depression Scale; HAMA, Hamilton anxiety scale; PDSS, Parkinson disease sleep scale; NMSQuest, non-motor symptoms questionnaire.*

## Discussion

To the best of our knowledge, this is the first study to assess the consistency and stability of the Jankovic, Schiess, and Kang classification systems (TD/indeterminate/PIGD and TD/mixed/AR, respectively) in *de novo* PD patients at a single center. We found that the TD/indeterminate/PIGD and the two TD/mixed/AR classifications have fair consistency, suggesting that a patient may be categorized as one subtype based on one classification system, but not according to another classification system. In addition, changes in subtype classification from baseline to 1-month follow-up indicated that there are other factors influencing the stability of motor subtype classification besides the course of the disease. Further, this is the first study to report that NMSs strongly affect the stability of the TD/indeterminate/PIGD classification.

Parkinson’s disease (PD) subtype identification is recognized as an essential research focus, as it may provide better paths for subtype-specific biomarkers and clinical trial designs, especially in terms of long-term prognosis (Sieber et al., 2014; Fereshtehnejad and Postuma, 2017). If PD subtypes predict a unique clinical course and are mutually independent and exclusive, they are prognostically relevant (Kotagal, 2016; Espay et al., 2017). However, due to the lack of reliable and effective subtype definition criteria, the PD motor subtype classifications lack these key elements. In the absence of these characteristics, it is not difficult to understand the inconsistency between PD motor subtype classification taxonomy and instability. Motor subtype classification systems follow a similar principle, classifying PD patients based on the ratio of two UPDRS subscores. However, different motor subtype classification systems use different UPDRS items and cutoff rates to define subtypes, suggesting that patients may be classified as tremor subtypes according to one motor subtype classification method but PIGD or AR subtypes according to another method. As a result, the consistency between different motor subtypes is fair. In a previous study, the Jankovic and Schiess classifications were found to poorly overlap in 103 *de novo* PD patients (Erro et al., 2019). By contrast, in the present study of 283 patients with *de novo* PD, we found that the Jankovic classification is fairly consistent with the two motor subtype classifications of TD/mixed/AR. Although differences in terms of sample size may lead to slightly different results, the overall consistency between different subtypes requires improvement. In addition, the instability of PD motor subtypes has been previously demonstrated (Simuni et al., 2016; Erro et al., 2019). The Parkinson’s Progression Biomarker Initiative (PPMI) consortium has decided to subdivide PD patients into TD and non-TD (PIGD and indeterminate) subtypes in order to reduce instability of the motor subtypes (Simuni et al., 2016). However, based on the Jankovic classification of similar cohorts of *de novo* PD patients, the prevalence of the TD phenotype varies from 29.3% as reported in the present study to 44.6% in an ongoing prospective research project (Erro et al., 2019) to 55.1% in the Deprenyl and Tocopherol Antioxidative Therapy of Parkinsonism (DATATOP) (Jankovic et al., 1990) cohort to 71.3% of the PPMI cohort (Simuni et al., 2016). Considering the large difference in the prevalence of TD subtypes, although stratification based solely on the variable of tremors will slightly increase stability, it may also increase the inconsistency of subtypes to a certain extent and generate confusion. Therefore, the consistency and stability of PD subtypes still need to be resolved, and powerful and validated criteria for PD subtype definition may be one of the most effective methods to achieve this.

The influence of DRT on the instability of PD motor subtypes warrants careful consideration. Although ‘practically defined OFF state’ is the conventional method for measuring the baseline degree of motor disability (Langston et al., 1992), the effect of DRT may be longer than the standard overnight flushing effect (Anderson and Nutt, 2011). In addition, its treatment effect on bradykinesia and rigidity is better than its effect on tremors. Therefore, a certain proportion of PIGD or AR patients were shifted into the TD group at 1-month follow-up. However, this does not explain the transfer of TD patients to other subtypes, which indicates that the instability of the motor subtype classification system does not depend mainly on DRT, which is consistent with previous reports (Simuni et al., 2016; Erro et al., 2019). There may be other potential factors explaining the observed instability. UPDRS provides operating standards for assessing the severity of symptoms; however, to some extent, it still depends on the evaluators. In addition, the TD subscores involved in defining motor subtypes in PD patients may fluctuate significantly at evaluations due to emotions such as worry and embarrassment. These factors may partially explain the observed instability of PD motor subtypes and the differences between studies.

Importantly, the stability of motor subtype classifications obtained through empirical clinical observation is greatly affected by disease duration. Recent studies have reported high variability in motor subtypes over 1, 2, and 4 years of follow-up in *de novo* PD cohorts (Simuni et al., 2016; Erro et al., 2019). Additionally, prospective and retrospective research of empirical motor subtype classification has shown that, over 8 years of follow-up, most TD subtypes eventually become the PIGD subtype (Alves et al., 2006; Josephs et al., 2006; Selikhova et al., 2009). However, a recent review suggested that disease course is insufficient to explain the shifts in motor subtypes (Nutt, 2016). In this context, the present study chose to reassess patients at one month to minimize the influence of disease duration so as to identify other potential variables that affect the stability of PD motor subtypes. Since the TD/indeterminate/PIGD classification had the worst stability among the three classifications, this classification was selected for further analysis of the factors influencing stability. Ultimately, we demonstrated that NMSs affect the stability of the TD/indeterminate/PIGD classification. Although PD motor subtype classification systems based on a single taxonomic factor ignore the non-motor features of PD, a longitudinal study confirmed that NMSs are significant indicators of prognosis and crucial characteristics of the definitions of PD subtypes (de Lau et al., 2014). Thus, inclusion of NMSs in the classification systems may result in more stable subtypes (Marras and Chaudhuri, 2016; Qian and Huang, 2019). Recently, novel PD clinical subtypes were identified using three critical NMSs (mild cognitive impairment, orthostatic hypotension, and rapid eye movement behavior disorder) and motor severity as key determinants in prospective cohorts (Fereshtehnejad et al., 2015), and then validated in the PPMI cohort with *de novo* PD patients (Fereshtehnejad et al., 2017). In light of the increasing importance of NMSs and the discovery of the novel subtype classification system, it may be time to redefine the entire motor subtype classification system of PD and its nomenclature.

When interpreting our findings, several limitations must be considered. First, our participants were *de novo* PD patients selected from a single center study. Thus, our cohort does not represent the entire PD patient population and our results may not be generalizable. However, differences in prevalence of TD subtypes were found in the similarly designed studies described above, which suggests that the type of recruitment may have a small influence on the results. Additionally, the sample size of this study is sufficiently large. Second, because the initial study design of the *de novo* PD cohort was based on annual follow-up to define biomarkers of PD diagnosis and progression, and the first month of follow-up data was collected due to increased attention to the instability of PD motor subtypes in recent years, there was a high drop-out rate for evaluation. However, there were no significant differences in baseline demographic and clinical features between patients with and without 1-month follow-up, suggesting that drop-out did not greatly affect the results. Third, although we compared demographic and clinical characteristics of patients with consistent and inconsistent subtypes, we did not account for variables such as genetics, environment, or other disease attributes. Fourth, *de novo* PD patients may still be mixed with patients with atypical parkinsonisms. Therefore, it is necessary to extend the longitudinal follow-up time to distinguish atypical parkinsonisms from PD.

## Conclusion

Fair consistency was observed between TD/indeterminate/PIGD and the two TD/mixed/AR classifications, indicating that patients may be divided into one subtype according to one classification system but not according to another classification system. Furthermore, for the first time, NMSs were found to influence the stability of TD/indeterminate/PIGD classification. Our findings strongly suggest that combining non-motor and conventional motor symptoms will improve the value of PD subtypes.

## Data Availability Statement

The original contributions presented in the study are included in the article/Supplementary Material, further inquiries can be directed to the corresponding author.

## Ethics Statement

The studies involving human participants were reviewed and approved by Ethics Committee of the Affiliated Brain Hospital of Nanjing Medical University. The patients/participants provided their written informed consent to participate in this study.

## Author Contributions

All authors listed have made a substantial, direct and intellectual contribution to the work, and approved it for publication.

## Conflict of Interest

The authors declare that the research was conducted in the absence of any commercial or financial relationships that could be construed as a potential conflict of interest.

## References

[B1] Alves G. Larsen J. P. Emre M. Wentzel-Larsen T. Aarsland D. (2006). Changes in motor subtype and risk for incident dementia in Parkinson’s disease. *Mov. Disord.* 21 1123–1130. 10.1002/mds.20897 16637023

[B2] Anderson E. Nutt J. (2011). The long-duration response to levodopa: phenomenology, potential mechanisms and clinical implications. *Parkinsonism Relat. Disord.* 17 587–592. 10.1016/j.parkreldis.2011.03.014 21530356

[B3] Armstrong M. J. Okun M. S. (2020). Diagnosis and treatment of Parkinson disease: a review. *JAMA* 323 548–560. 10.1001/jama.2019.22360 32044947

[B4] Calne D. B. (1989). Is “Parkinson’s disease” one disease? *J. Neurol. Neurosurg. Psychiatry* 52(Suppl) 18–21. 10.1136/jnnp.52.suppl.18 2666575 PMC1033305

[B5] Chaudhuri K. R. Martinez-Martin P. Schapira A. H. Stocchi F. Sethi K. Odin P. (2006). International multicenter pilot study of the first comprehensive self-completed nonmotor symptoms questionnaire for Parkinson’s disease: the NMSQuest study. *Mov. Disord.* 21 916–923. 10.1002/mds.20844 16547944

[B6] Choi S. M. Kim B. C. Cho B. H. Kang K. W. Choi K. H. Kim J. T. (2018). Comparison of two motor subtype classifications in de novo Parkinson’s disease. *Parkinsonism Relat. Disord.* 54 74–78. 10.1016/j.parkreldis.2018.04.021 29703644

[B7] de Lau L. M. Verbaan D. van Rooden S. M. Marinus J. van Hilten J. J. (2014). Relation of clinical subtypes in Parkinson’s disease with survival. *Mov. Disord.* 29 150–151. 10.1002/mds.25652 24038593

[B8] Eisinger R. S. Hess C. W. Martinez-Ramirez D. Almeida L. Foote K. D. Okun M. S. (2017). Motor subtype changes in early Parkinson’s disease. *Parkinsonism Relat. Disord.* 43 67–72. 10.1016/j.parkreldis.2017.07.018 28754232 PMC5842811

[B9] Eisinger R. S. Martinez-Ramirez D. Ramirez-Zamora A. Hess C. W. Almeida L. Okun M. S. (2020). Parkinson’s disease motor subtype changes during 20 years of follow-up. *Parkinsonism Relat. Disord.* 76 104–107. 10.1016/j.parkreldis.2019.05.024 31129020 PMC6980332

[B10] Erro R. Picillo M. Amboni M. Savastano R. Scannapieco S. Cuoco S. (2019). Comparing postural instability and gait disorder and akinetic-rigid subtyping of Parkinson disease and their stability over time. *Eur. J. Neurol.* 26 1212–1218. 10.1111/ene.13968 30985953

[B11] Espay A. J. Schwarzschild M. A. Tanner C. M. Fernandez H. H. Simon D. K. Leverenz J. B. (2017). Biomarker-driven phenotyping in Parkinson’s disease: a translational missing link in disease-modifying clinical trials. *Mov. Disord.* 32 319–324. 10.1002/mds.26913 28233927 PMC5359057

[B12] Fereshtehnejad S. M. Postuma R. B. (2017). Subtypes of Parkinson’s disease: what do they tell us about disease progression? *Curr. Neurol. Neurosci. Rep.* 17:34. 10.1007/s11910-017-0738-x 28324303

[B13] Fereshtehnejad S. M. Romenets S. R. Anang J. B. Latreille V. Gagnon J. F. Postuma R. B. (2015). New clinical subtypes of Parkinson disease and their longitudinal progression: a prospective cohort comparison with other phenotypes. *JAMA Neurol.* 72 863–873. 10.1001/jamaneurol.2015.0703 26076039

[B14] Fereshtehnejad S. M. Zeighami Y. Dagher A. Postuma R. B. (2017). Clinical criteria for subtyping Parkinson’s disease: biomarkers and longitudinal progression. *Brain* 140 1959–1976. 10.1093/brain/awx118 28549077

[B15] Gibb W. R. Lees A. J. (1988). The relevance of the Lewy body to the pathogenesis of idiopathic Parkinson’s disease. *J. Neurol. Neurosurg. Psychiatry* 51 745–752. 10.1136/jnnp.51.6.745 2841426 PMC1033142

[B16] Graham J. M. Sagar H. J. (1999). A data-driven approach to the study of heterogeneity in idiopathic Parkinson’s disease: identification of three distinct subtypes. *Mov. Disord.* 14 10–20. 10.1002/1531-8257(199901)14:1<10::aid-mds1005<3.0.co;2-4 9918339

[B17] Guan X. Zeng Q. Guo T. Wang J. Xuan M. Gu Q. (2017). Disrupted functional connectivity of basal ganglia across tremor-dominant and akinetic/rigid-dominant Parkinson’s disease. *Front. Aging Neurosci.* 9:360. 10.3389/fnagi.2017.00360 29163141 PMC5673841

[B18] Jankovic J. McDermott M. Carter J. Gauthier S. Goetz C. Golbe L. (1990). Variable expression of Parkinson’s disease: a base-line analysis of the DATATOP cohort. the Parkinson study group. *Neurology* 40 1529–1534. 10.1212/wnl.40.10.1529 2215943

[B19] Josephs K. A. Matsumoto J. Y. Ahlskog J. E. (2006). Benign tremulous parkinsonism. *Arch. Neurol.* 63 354–357. 10.1001/archneur.63.3.354 16533962

[B20] Kang G. A. Bronstein J. M. Masterman D. L. Redelings M. Crum J. A. Ritz B. (2005). Clinical characteristics in early Parkinson’s disease in a central California population-based study. *Mov. Disord.* 20 1133–1142. 10.1002/mds.20513 15954133 PMC3643967

[B21] Kotagal V. (2016). Is PIGD a legitimate motor subtype in Parkinson disease? *Ann. Clin. Transl. Neurol.* 3 473–477. 10.1002/acn3.312 27547776 PMC4892002

[B22] Landis J. R. Koch G. G. (1977). The measurement of observer agreement for categorical data. *Biometrics* 33 159–174. 10.2307/2529310 843571

[B23] Langston J. W. Widner H. Goetz C. G. Brooks D. Fahn S. Freeman T. (1992). Core assessment program for intracerebral transplantations (CAPIT). *Mov. Disord.* 7 2–13. 10.1002/mds.870070103 1557062

[B24] Lian T. H. Guo P. Zuo L. J. Hu Y. Yu S. Y. Yu Q. J. (2019). Tremor-dominant in Parkinson disease: the relevance to iron metabolism and inflammation. *Front. Neurosci.* 13:255. 10.3389/fnins.2019.00255 30971879 PMC6445850

[B25] Marras C. Chaudhuri K. R. (2016). Nonmotor features of Parkinson’s disease subtypes. *Mov. Disord.* 31 1095–1102. 10.1002/mds.26510 26861861

[B26] Nutt J. G. (2016). Motor subtype in Parkinson’s disease: different disorders or different stages of disease? *Mov. Disord.* 31 957–961. 10.1002/mds.26657 27226220

[B27] Polychronis S. Niccolini F. Pagano G. Yousaf T. Politis M. (2019). Speech difficulties in early de novo patients with Parkinson’s disease. *Parkinsonism Relat. Disord.* 64 256–261. 10.1016/j.parkreldis.2019.04.026 31078401

[B28] Qian E. Huang Y. (2019). Subtyping of Parkinson’s disease – where are we up to? *Aging Dis.* 10 1130–1139. 10.14336/ad.2019.0112 31595207 PMC6764738

[B29] Ren J. Hua P. Li Y. Pan C. Yan L. Yu C. (2020a). Comparison of three motor subtype classifications in de novo Parkinson’s disease patients. *Front. Neurol.* 11:601225. 10.3389/fneur.2020.601225 PMC7785849 33424750

[B30] Ren J. Hua P. Pan C. Li Y. Zhang L. Zhang W. (2020b). Non-motor symptoms of the postural instability and gait difficulty subtype in de novo Parkinson’s disease patients: a cross-sectional study in a single center. *Neuropsychiatr. Dis. Treat.* 16 2605–2612. 10.2147/ndt.S280960 33173298 PMC7646450

[B31] Schiess M. C. Zheng H. Soukup V. M. Bonnen J. G. Nauta H. J. (2000). Parkinson’s disease subtypes: clinical classification and ventricular cerebrospinal fluid analysis. *Parkinsonism Relat. Disord.* 6 69–76. 10.1016/s1353-8020(99)00051-6 10699387

[B32] Selikhova M. Williams D. R. Kempster P. A. Holton J. L. Revesz T. Lees A. J. (2009). A clinico-pathological study of subtypes in Parkinson’s disease. *Brain* 132(Pt 11) 2947–2957. 10.1093/brain/awp234 19759203

[B33] Sieber B. A. Landis S. Koroshetz W. Bateman R. Siderowf A. Galpern W. R. (2014). Prioritized research recommendations from the National institute of neurological disorders and stroke Parkinson’s disease 2014 conference. *Ann. Neurol.* 76 469–472. 10.1002/ana.24261 25164235 PMC5736367

[B34] Simuni T. Caspell-Garcia C. Coffey C. Lasch S. Tanner C. Marek K. (2016). How stable are Parkinson’s disease subtypes in de novo patients: analysis of the PPMI cohort? *Parkinsonism Relat. Disord.* 28 62–67. 10.1016/j.parkreldis.2016.04.027 27132498

[B35] Tomlinson C. L. Stowe R. Patel S. Rick C. Gray R. Clarke C. E. (2010). Systematic review of levodopa dose equivalency reporting in Parkinson’s disease. *Mov. Disord.* 25 2649–2653. 10.1002/mds.23429 21069833

[B36] van Rooden S. M. Heiser W. J. Kok J. N. Verbaan D. van Hilten J. J. Marinus J. (2010). The identification of Parkinson’s disease subtypes using cluster analysis: a systematic review. *Mov. Disord.* 25 969–978. 10.1002/mds.23116 20535823

