# Supplementary material for: Consistency and Stability of Motor Subtype Classifications in Patients With de novo Parkinson’s Disease
Source: Front Neurosci. 2021 Mar 1;15:637896. doi: 10.3389/fnins.2021.637896 (PMC7957002; doi:10.3389/fnins.2021.637896)
Supplement: Supplementary file 1 [file Table_1.pdf]

Supplementary Table 1

Demographic and clinical characteristics of the 79 PD patients at 1-month follow-up.

**Variables**	**1-month follow-up (n=79)**
Age (years)	60.5 ± 7.3
Gender (male)	39 (49.4%)
Formal education (years)	9.6 ± 3.7
Age at onset (years)	58.5 ± 7.4
Duration of symptom onset (years)	2.1 ± 1.5
UPDRS part II	6.5 ± 3.4
UPDRS part III	18.4 ± 10.2
Modified H-Y stage	1.5 ± 0.5
MMSE	27.7 ± 2.8
MoCA	24.1 ± 4.0
HAMD	8.0 ± 6.4
HAMA	6.2 ± 4.7
PDSS	125.7 ± 25.8
NMSQuest	7.2 ± 4.4
LEDD	74.5 ± 4.2

Data are presented as the mean ± SD and n (%). UPDRS, Unified Parkinson's Disease Rating Scale; H-Y, Hoehn and Yahr; MMSE, Mini-Mental State Examination; MoCA, Montreal Cognitive Assessment; HAMD, Hamilton Depression Scale; HAMA, Hamilton Anxiety Scale; PDSS, Parkinson Disease Sleep Scale; NMSQuest, Non-motor symptoms Questionnaire; LEDD, Levodopa Equivalent Daily Dose.
